# Impact of Hydrogel Stiffness on Differentiation of Human Adipose-Derived Stem Cell Microspheroids

**DOI:** 10.1089/ten.tea.2018.0237

**Published:** 2019-10-04

**Authors:** Sara Žigon-Branc, Marica Markovic, Jasper Van Hoorick, Sandra Van Vlierberghe, Peter Dubruel, Elise Zerobin, Stefan Baudis, Aleksandr Ovsianikov

**Affiliations:** ^1^ Institute of Materials Science and Technology, Technische Universität Wien (TU Wien), Vienna, Austria.; ^2^ Austrian Cluster for Tissue Regeneration, Austria.; ^3^ Department of Organic and Macromolecular Chemistry, Polymer Chemistry and Biomaterials Group, Centre of Macromolecular Chemistry, Ghent University, Ghent, Belgium.; ^4^ Brussels Photonics, Department of Applied Physics and Photonics, Vrije Universiteit Brussel and Flanders Make, Elsene, Belgium.; ^5^ Division of Macromolecular Chemistry, Institute of Applied Synthetic Chemistry, Technische Universität Wien (TU Wien), Vienna, Austria.

**Keywords:** adult stem cells, cell encapsulation, hydrogels, cell differentiation, cartilage, bone

## Abstract

Hydrogels represent an attractive material platform for realization of three-dimensional (3D) tissue-engineered constructs, as they have tunable mechanical properties, are compatible with different types of cells, and resemble elements found in natural extracellular matrices. So far, numerous hydrogel-cartilage/bone tissue engineering (TE)-related studies were performed by utilizing a single cell encapsulation approach. Although multicellular spheroid cultures exhibit advantageous properties for cartilage or bone TE, the chondrogenic or osteogenic differentiation potential of stem cell microspheroids within hydrogels has not been investigated much. This study explores, for the first time, how stiffness of gelatin-based hydrogels (having a storage modulus of 538, 3584, or 7263 Pa) affects proliferation and differentiation of microspheroids formed from telomerase-immortalized human adipose-derived stem cells (hASC/hTERT). Confocal microscopy indicates that all tested hydrogels supported cell viability during their 3- to 5-week culture period in the control, chondrogenic, or osteogenic medium. Although in the softer hydrogels cells from neighboring microspheroids started outgrowing and interconnecting within a few days, their protrusion was slower or limited in stiffer hydrogels or those cultured in chondrogenic medium, respectively. High expressions of chondrogenic markers (*SOX9*, *ACAN*, *COL2A1*), detected in all tested hydrogels, proved that the chondrogenic differentiation of hASC/hTERT microspheroids was very successful, especially in the two softer hydrogels, where superior cartilage-specific properties were confirmed by Alcian blue staining. These chondrogenically induced samples also expressed *COL10A1*, a marker of chondrocyte hypertrophy. Interestingly, the hydrogel itself (with no differentiation medium) showed a slight chondrogenic induction. Regardless of the hydrogel stiffness, in the samples stimulated with osteogenic medium, the expression of selected markers *RUNX2*, *BGLAP*, *ALPL*, and *COL1A1* was not conclusive. Nevertheless, the von Kossa staining confirmed the presence of calcium deposits in osteogenically stimulated samples in the two softer hydrogels, suggesting that these also favor osteogenesis. This observation was also confirmed by Alizarin red quantification assay, with which higher amounts of calcium were detected in the osteogenically induced hydrogels than in their controls. The presented data indicate that the encapsulation of adipose-derived stem cell microspheroids in gelatin-based hydrogels show promising potential for future applications in cartilage or bone TE.

**Impact Statement:**

Osteochondral defects represent one of the leading causes of disability in the world. Although numerous tissue engineering (TE) approaches have shown success in cartilage and bone tissue regeneration, achieving native-like characteristics of these tissues remains challenging. This study demonstrates that in the presence of a corresponding differentiation medium, gelatin-based hydrogels support moderate osteogenic and excellent chondrogenic differentiation of photo-encapsulated human adipose-derived stem cell microspheroids, the extent of which depends on hydrogel stiffness. Because photosensitive hydrogels are a convenient material platform for creating stiffness gradients in three dimensions, the presented microspheroid-hydrogel encapsulation strategy holds promise for future strategies of cartilage or bone TE.

## Introduction

Hydrogels are among the most promising materials for three-dimensional (3D) cell culture, as they mimic important properties of extracellular matrices (ECMs), have similar mechanics to many soft tissues, and support cell adhesion.^1–3^ Besides giving structural support, ECM regulates cell behavior and importantly affects tissue formation and function.^4^

In the last two decades methacrylamide-modified gelatin (Gel-MOD) has shown great potential for various bioengineering and biofabrication approaches because of its cytocompatibility and tunability.^5–7^ In addition to light dose, cross-linking of Gel-MOD can be altered and controlled through a variation of the degree of methacrylation or material concentration yielding a range of different mechanical properties.^8,9^ The initial amount of photopolymerizable materials is directly correlated to the network density and the stiffness of the cross-linked hydrogel.

Previous reports show that varying the stiffness of two-dimensional (2D) or 3D substrates significantly influence stem cell migration, proliferation, and differentiation.^10^ So far, the impact of Gel-MOD stiffness toward osteo- or chondrogenic propagation was addressed in studies of photo-encapsulation of single cell suspensions of bovine and porcine chondrocytes, human or rat mesenchymal stem cells (MSCs), and a human osteosarcoma cell line MG63. Although researchers reported a supporting effect of softer compositions (i.e. ≤10% [w/v]) of the cross-linked Gel-MOD on osteo- or chondrogenic phenotypes of selected cells, because of different culturing conditions used it is impossible to compare the results of these studies. Moreover, the effect of Gel-MOD stiffness to induce chondrogenic or osteogenic differentiation of photo-encapsulated microspheroid tissues (i.e. microspheroids) *per se* or in conjunction with an adequate differentiation medium has not yet been addressed.

Multicellular spheroids are well-known “building blocks” in the field of tissue engineering (TE).^11–23^ It is established that microspheroid cultures promote and support chondrogenic and osteogenic differentiation of MSCs.^21,24,25^ Compared with cells seeded on a matrix, spheroids composed of chondrocytes or chondrogenically differentiated MSCs or adipose-derived stem cells (ASCs) achieved histological, biochemical, and biomechanical characteristics close to native cartilage.^26–29^ In addition, condensation of MSCs represents one of the earliest phases of the *in vivo* cartilage development, an important aspect in cartilage TE.^28^ Compared with monolayer cultures, osteogenic differentiation proceeds faster in microspheroids as the cell architecture changes, enhancing the production of a bone-like ECM.^14,16,17,19,23^

Although the fusion of multiple spheroids enables generation of larger continuous constructs, the need for huge amounts of cells represents a limiting factor. In this regard, a combination of scaffold-free and scaffold-based TE approaches could result in an optimal tissue construct, possibly by using microspheroids, enhancing the seeding efficiency of the hydrogel and consequently accelerating tissue formation.

In this study, the impact of varying stiffness properties of Gel-MOD on the osteo- and chondrogenic differentiation potential of photo-encapsulated hASC/hTERT microspheroids was investigated using confocal microscopy, gene expression analysis, histology, and calcium quantification. Three different Gel-MOD hydrogels were prepared by adjusting the material concentration, followed by photo-encapsulation of the microspheroids and their incubation in a selected differentiation medium. The mechanical properties of the hydrogels were analyzed using rheology.

## Materials and Methods

Unless otherwise stated, reagents were purchased from Sigma-Aldrich, Germany.

### Stem cell culture and encapsulation in Gel-MOD

Immortalized human adipose-derived mesenchymal stem cells (hASC/hTERT) (Evercyte, Austria) were expanded using EGM™-2 BulletKit™ medium (Lonza, Switzerland) supplemented with 10% (v/v) newborn calf serum (NBCS) (Gibco, New Zealand) and maintained at standard culturing conditions (37°C, 5% CO_2_, humidified atmosphere). Medium was refreshed three times per week and hASC/hTERT were subcultured after reaching 80% confluence.

To obtain microspheroids, 256,000 cells (passage 7) were seeded on 256-well agarose MicroTissues^®^ 3D Petri Dishes^®^ (Sigma-Aldrich, MO) according to the manufacturer's protocol in control medium (high glucose Dulbecco's modified Eagle medium [HG-DMEM; Gibco, United Kingdom] supplemented with 10% [v/v] NBCS and 1% [v/v] penicillin [10,000 U]–streptomycin [10 mg/mL] solution [P/S]) and incubated for 48 h at standard culturing conditions.

The formed microspheroids were resuspended in either 5%, 7.5%, or 10% (wt%) methacrylated gelatin (Gel-MOD) solution in control medium containing 0.6 mM photoinitiator [lithium (2,4,6-trimethylbenzoyl)-phenylphosphinate (Li-TPO)]. Gel-MOD (with a degree of substitution of 63%) and Li-TPO solutions were prepared as reported.^30–32^ Subsequently, 30 μL of the Gel-MOD suspension, containing ∼81 spheroids, was dispensed on methacrylated 35 mm high μ-dishes or four-well μ-slide chambers (ibidi, Germany). The methacrylation was carried out as already described.^33^ Samples were exposed to 25 mW/cm^2^ UV-A light (LITE-Box G136 365 nm; NK-OPTIK, Germany) for 10 min to induce hydrogel cross-linking. Afterwards 0.5 mL of control medium per gel clot was added and the dishes were transferred to the incubator.

### Chondrogenic and osteogenic differentiation

After a 24 h incubation of hydrogel clots in control medium, the latter was replaced with control, chondrogenic, or osteogenic medium. Chondrogenic medium consisted of HG-DMEM supplemented with 1% (v/v) insulin–transferrin–selenium supplement (Gibco, United Kingdom), 1% (v/v) of P/S, 1% (v/v) 1 M HEPES buffer (Mediatech, VA), 0.1 mg/mL sodium pyruvate, 50 μg/mL l-proline, 50 μg/mL ascorbic acid 2-phosphate, 100 nM dexamethasone, and 10 ng/mL of human transforming growth factor β_3_ (Peprotech, NY) and human bone morphogenic protein 6 (R&D, MN). Osteogenic medium was composed of HG-DMEM supplemented with 10% (v/v) NBCS, 4 mM l-glutamine, 1% (v/v) P/S, 10 nM dexamethasone, 150 μM ascorbic acid 2-phosphate, 10 mM β-glycerophosphate, and 10 nM 1,25-vitamin D3. Hydrogels containing microspheroids were incubated for 3 weeks in osteogenic medium and 5 weeks in control or chondrogenic medium, with medium refreshment three times per week.

### Cell viability

Cell viability was determined using a Live/Dead^®^ assay (Invitrogen, OR). After rinsing the hydrogels three times with phosphate-buffered saline (PBS), these were incubated in 0.2 μM calcein-AM (live stain) and 0.6 μM propidium iodide (dead stain) in PBS for 30 min at 37°C. The viability of cells was monitored weekly using a confocal laser scanning microscope LSM 700 (Zeiss, Germany). Viable cells emitted green fluorescence at excitation/emission set at 488/530 nm, whereas nuclei of dead cells appeared red at 530/580 nm.

### Quantitative real-time polymerase chain reaction

After 3 weeks of cell differentiation, six gel clots per treatment group were merged and total RNA was isolated using RNeasy^®^ Plus Universal Mini Kit (Qiagen, Germany) according to manufacturer's instructions. RNA concentrations were measured using a Synergy H1 spectrophotometer (BioTek, VT). From each sample 1 μg of RNA was isolated, treated with AccuRT Genomic DNA Removal Kit (ABM, Canada), and reverse transcribed into cDNA using 5X All-In-One RT MasterMix (ABM). Using a CFX Connect Real-Time System (BioRad, VT), quantitative real-time polymerase chain reaction (qPCR) was performed according to the BioRad PrimePCR_Assay_Quick_Guide_D101868_VerB. Primer mixes used in qPCR are given in Table 1.

**Table 1.** List of Genes Used in Quantitative Real-Time Polymerase Chain Reaction Experiments

*Gene symbol*	*Gene name*	*Producer, assay ID*
*ALPL*	Alkaline phosphatase	BioRad, qHsaCID0010031
*ACAN*	Aggrecan	BioRad, qHsaCID0008122
*BGLAP*	Osteocalcin	BioRad, qHsaCED0038437
*COL1A1*	Collagen type I, alpha 1	BioRad, qHsaCED0043248
*COL2A1*	Collagen type II, alpha 1	BioRad, qHsa CED0001057
*COL10A1*	Collagen type X, alpha 1	BioRad, qHsa CID0007356
*HPRT1*	Hypoxanthine-guanine phosphoribosyltransferase	Qiagen, QT00059066
*RUNX2*	Runt-related transcriptor factor 2	BioRad, qHsaCID0006726
*SOX9*	SRY (sex determining region Y)—box 9	BioRad, qHsaCED0044083

In total 40 cycles of qPCR were performed as follows: activation (30 s at 95°C), denaturation (15 s at 95°C), and annealing and extension (15 s at 60°C). Data were processed using CFX Manager Version 3.1 (BioRad) and relative gene expression (RQ) was calculated with the formula RQ = 2^–ΔΔCq^.^34^ For each tested group four cDNA samples were obtained and for each qPCR was performed in duplicate. The calculated ΔCq values were normalized to the ΔCq values of 2D controls (cells grown in 2D before encapsulation). For low expressed genes, a cutoff value of Cq ≥35 was used.

### Histology and calcium quantification analyses

After a 3- or 5-week differentiation of hASC/hTERT in different Gel-MOD hydrogels, these were washed with PBS and fixed overnight in Roti^®^Histofix 4% (Carl Roth, Germany) at 4°C. Hydrogels were either embedded in paraffin blocks and processed at the HistoPathology Department (Vienna BioCenter Core Facilities GmbH, Austria) or used in a calcium quantification assay (Supplementary Data S1).

### Mechanical testing of Gel-MOD hydrogels

Mechanical tests were performed using rheology as previously reported.^35^ In brief, UV cross-linked Gel-MOD sheets (1 mm thick) were obtained by film casting starting from the hydrogel precursor solutions as described previously. The precursor solutions were injected between 4 mm thick clear glass slides and cross-linked as in cell encapsulation experiments. The hydrogel sheets were incubated in PBS at 37°C to induce equilibrium swelling. Subsequently, hydrogel discs (diameter = 14 mm) were punched from the sheets and placed between the plates of a plate-plate rheometer at 37°C (Anton Paar Physica MCR-301; Anton Paar, Belgium). A frequency sweep (0.01–10 Hz at 0.1% strain) and an amplitude scan (0.01–10% at 1 Hz) were performed keeping a constant normal force of 1 N, to ensure proper contact between the sample and the plates. During the measurements, samples were immersed in PBS to prevent drying. The average storage (*G*′) and loss (*G*″) moduli were determined within the linear viscoelastic region of the hydrogels.

### Network density calculations

Network density calculations are reported in detail in Supplementary Data S2. To obtain the swelling ratio, the mass of the hydrogel discs (diameter = 14 mm) was determined at equilibrium swelling (*m_w_*) (in PBS at 37°C), after gently removing surface water with tissue paper. Next, samples were freeze-dried and their dry mass was determined (*m_d_*). The swelling ratio (*q*) was calculated as: *q* = *m_w_*/*m_d_*.

### Data analysis

All results are presented as mean ± standard deviation (SD). In our study, the criterion RQ ≥ ±2 represented significant changes in gene expression.^36,37^ In addition, one-way analysis of variance (ANOVA) with Tukey's *post hoc* test was used to evaluate statistical differences between samples. Significance was assumed for *p* < 0.05, *p* < 0.01, and *p* < 0.001 values, shown in figures as *, **, and ***, respectively. Data analyses were carried out in GraphPad Prism^®^ 5.0. (GraphPad Software, Inc., San Diego, CA).

## Results

### Viability and morphology of encapsulated cells

**FIG. 1.**
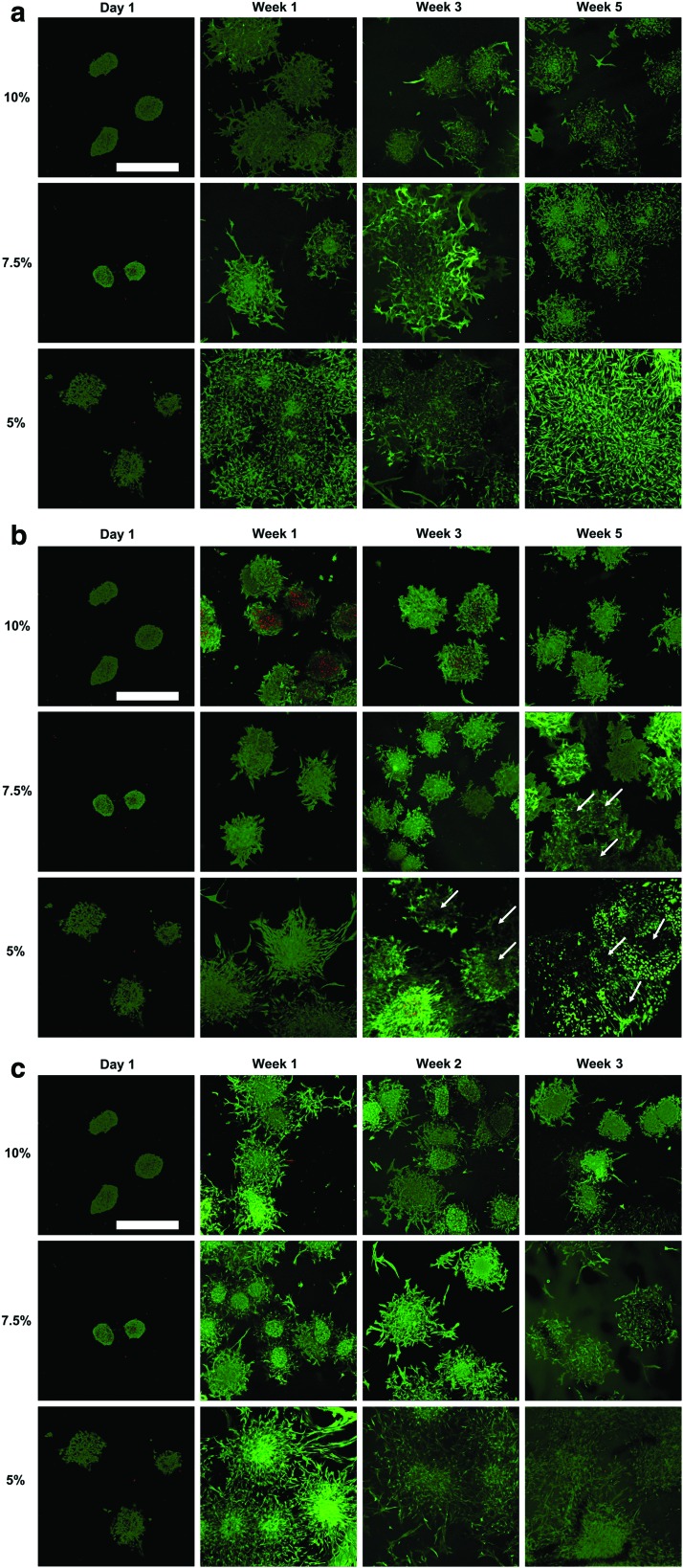
Live/dead staining of encapsulated hASC/hTERT in 5%, 7.5%, and 10% Gel-MOD hydrogels, cultured in **(a)** control or **(b)** chondrogenic medium for a period of 3–5 weeks, or **(c)** osteogenic medium for a period of 1–3 weeks. The viability of cells was also verified 1 day after their encapsulation, before starting differentiation. Viable cells emitted *green* fluorescence, whereas the nuclei of dead cells appeared *red*. *White arrows* indicate “voids” that appeared in the microspheroids. Scale bars = 500 μm. hASC/hTERT, telomerase-immortalized human adipose-derived stem cells. Color images are available online.

Following a 24 h encapsulation of microspheroids, the cells were viable in all tested hydrogels and started sprouting in the 5% Gel-MOD (Fig. 1a). After 1 week, cells cultured in the control medium started sprouting in 7.5% and 10% gels, whereas in the 5% hydrogel cells from neighboring microspheroids started interconnecting. In all hydrogels, the cells stretched and became spindle shaped. At week 1, cell outgrowth from the chondrogenically induced microspheroids was less pronounced than in the control medium (Fig. 1b). In the 10% hydrogel some microspheroid cores appeared partially necrotic, but after 3–5 weeks of chondrogenic differentiation, necrotic cores were no longer visible. The spheroid viability remained preserved, cell protrusion was limited, and cells became rounder. In the 5% and 7.5% gels cell sprouting was stronger, cells acquired a round morphology, and “voids” around microspheroid core areas were observed. Although after a 1-week microspheroidal culture in osteogenic medium cell sprouting reached a similar extent as in the control medium, later on cell protrusion was (except for the 5% hydrogel) weaker in stiffer hydrogels (Fig. 1c). Moreover, the cell shape was similar to the control. Regardless of the culture medium used, cells remained viable throughout the experiment.

### Impact of different Gel-MOD stiffness properties on gene expression

**FIG. 2.**
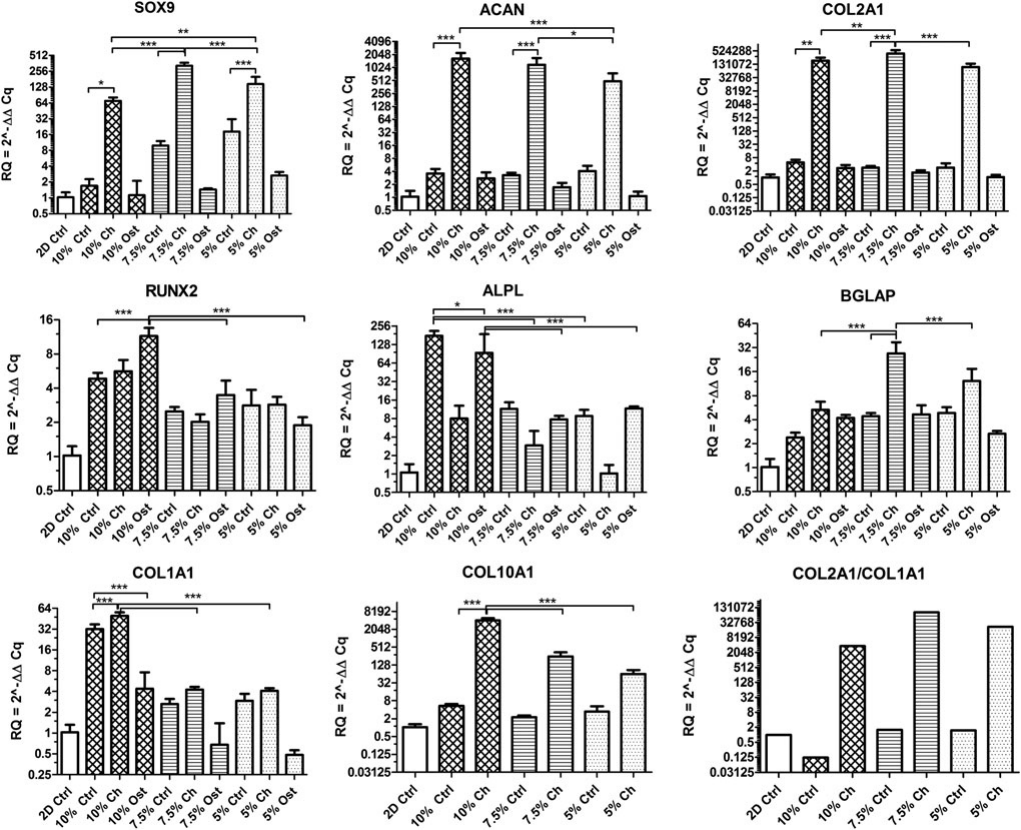
Gene expression analysis of encapsulated hASC/hTERT in 5%, 7.5%, and 10% Gel-MOD hydrogels after a 3-week differentiation in control (Ctrl), chondrogenic (Ch), or osteogenic medium (Ost). Mean of relative expression (RQ) ± SD is presented, number of biological repetitions = 4. Value 1 represents basal gene expression (2D Ctrl) and RQ values ≥2 represent significant changes in gene expression. In addition, one-way ANOVA with Tukey's *post hoc* test was used to compare RQ values (*n* = 4); significance was assumed for *p* < 0.05, *p* < 0.01, and *p* < 0.001 values, shown in figures as *, ** or ***, respectively. *Bottom right corner*: differentiation index (*COL2A1/COL1A1* ratio) calculated for control (Ctrl) and chondrogenically (Ch) differentiated samples. Note differences in scales. ANOVA, analysis of variance; SD, standard deviation.

The impact of a 3-week long differentiation of hASC/hTERT microspheroids encapsulated in selected hydrogels was verified by qPCR (Fig. 2). Compared to undifferentiated 2D controls, gene expressions (RQ values) of *SOX9*, *ACAN*, *COL2A1*, and *COL10A1* were already more than two times higher in almost all 3D controls. Moreover, when the samples were cultured in chondrogenic medium, the gene expressions increased tremendously—more than 70 times for *SOX9*, 430 times for *ACAN*, 88,500 times for *COL2A1*, and 63 times for *COL10A1*. Among all hydrogels the highest expressions of *SOX9*, *ACAN*, and *COL2A1* were observed within the 7.5% gel. The expression of *COL1A1* was slightly increased in 5% and 7.5% Gel-MOD control and chondrogenically differentiated samples and was 10 times higher in both conditions in 10% gel. Nevertheless, except for the 10% Gel-MOD control, the differentiation index (i.e. *COL2A1/COL1A1* ratio) was positive in 5% and 7.5% Gel-MOD controls and exceptionally high in all chondrogenically differentiated samples. Chondrogenically differentiated samples also expressed moderate to high levels of *COL10A*, which correlated to hydrogel stiffness.

Compared to the 2D control, expressions of *RUNX2*, *BGLAP*, and *ALPL* obtained from osteogenically differentiated microspheroids were slightly to moderately increased in all three hydrogels. However, compared to their corresponding 3D controls, the expression of *RUNX2* was similar in 5% and 7.5% hydrogels and two times lower in the 10% control. Similar expression profiles among 3D controls and corresponsive osteogenically differentiated samples were also observed for *BGLAP* and *ALPL*, with the exception of the 10% gel, where *ALPL* was almost two times higher in the 3D control than in the osteogenically differentiated group. However, because of a very high SD of the latter, this result is inconclusive. A similar expression trend was observed for *COL1A1*, but with a 10-fold difference. Nonetheless, compared with 2D or 3D corresponding controls, the expression of *COL1A1* was downregulated in 5% and 7.5% hydrogels.

### Chondrogenic differentiation of encapsulated microspheroids

**FIG. 3.**
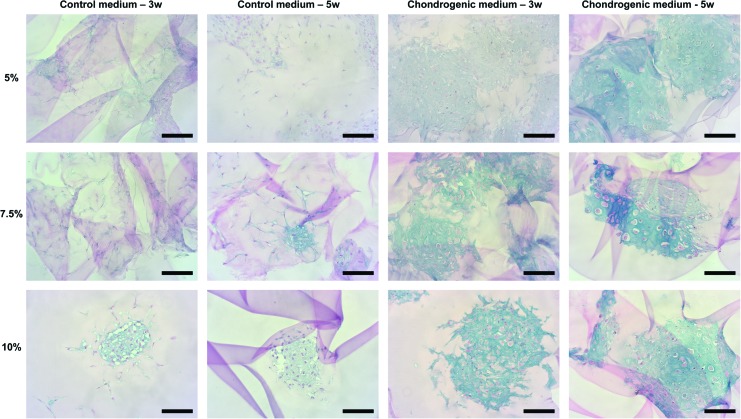
Glycosaminoglycan formation detected with Alcian blue staining after hASC/hTERT microspheroid encapsulation in 5%, 7.5%, or 10% Gel-MOD hydrogels and a 3- or 5-week differentiation in control or chondrogenic medium. Scale bars = 100 μm. Color images are available online.

After culturing the encapsulated hASC/hTERT microspheroids in chondrogenic medium for 3 or 5 weeks, formation of glycosaminoglycans (GAGs) was histologically confirmed in all hydrogels (Fig. 3 and Supplementary Fig. S1). The intensities of the Alcian blue dye (bound to GAGs) and the morphological appearances of the formed cartilaginous-like tissues were stronger in samples cultured for 5 weeks versus 3 weeks, showing a superior tissue-specific organization in 5% and 7.5% hydrogels. Interestingly, a weak-positive staining was also observed in 7.5% and 10% Gel-MOD control (undifferentiated) samples (Fig. 3).

### Calcium deposition

**FIG. 4.**
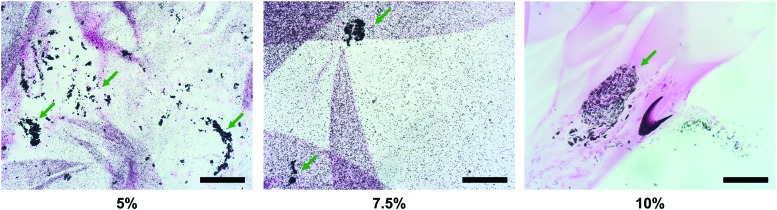
Visualization of calcium mineralization (*black deposits*) using von Kossa staining after hASC/hTERT microspheroid encapsulation in 5%, 7.5%, or 10% Gel-MOD hydrogels and a 3-week differentiation in osteogenic medium. *Green arrows* indicate stronger mineralization in close proximity to the encapsulated microspheroids. Scale bars = 100 μm. Color images are available online.

The von Kossa staining of microspheroids encapsulated in 5–10% hydrogels, cultured for 3–5 weeks in control or chondrogenic medium, revealed no mineral deposits. Identical results were obtained after a 3-week incubation of cell-free hydrogels in all three culture media (Supplementary Fig. S1). However, when the encapsulated microspheroids were cultured in osteogenic medium for 3 weeks, almost uniformly distributed mineral deposits were observed in 5% and 7.5% gel/tissue cross-sections (Fig. 4). Moreover, a stronger mineralization was detected in close proximity to the encapsulated microspheroids. In contrast, the mineral content of 10% gels was much weaker. Regardless of the hydrogel stiffness, microspheroids cultured for 3 weeks in osteogenic medium produced two to three times more calcium than controls (results not given). Samples cultured in chondrogenic or control medium contained similar calcium quantities, whereas no calcium was detected in the cell-free hydrogels.

### Mechanical properties and network density calculations

**FIG. 5.**
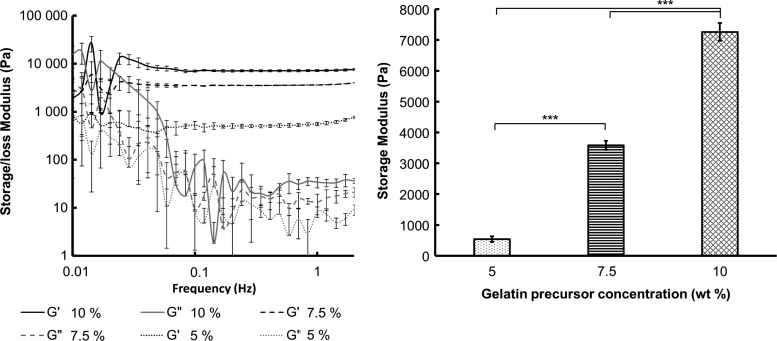
Rheological analysis of the hydrogel films. Frequency sweep (*n* = 3) performed on the hydrogel films with different Gel-MOD concentrations in equilibrium swollen state (*left panel*). Average storage modulus *G*′ (Pa) extrapolated from the linear viscoelastic region including SD (*n* = 3) (*right panel*). One-way ANOVA with Tukey's *post hoc* test was used to determine statistical differences among the measured values (*n* = 3); *** indicates statistical significance at *p* < 0.001.

The storage modulus *G*′, which can be considered a measure of hydrogel stiffness, at 5%, 7.5%, and 10% Gel-MOD concentrations corresponded to 538 ± 91, 3584 ± 146, and 7263 ± 287 Pa, respectively (Fig. 5). The Gel-MOD content prior cross-linking drastically influenced the final mechanical properties of the hydrogels. The same was observed in the network density calculations (based on molecular weight [Supplementary Fig. S2], water uptake capacity and rheological properties), where lower initial concentrations lead to looser networks and vice versa (Table 2).

**Table 2.** Overview of Measured Mass Swelling Ratio, Storage Modulus (*G*′) via Rheology and Properties Calculated Using the Rubber Elasticity Theory

*Concentration (wt %)*	*Mass swelling ratio (*q*)*	G′ *at 37°C (Pa)*	Q	Mc *(g/mol)*	ξ *(Å)*	ρ *( × 10^−4^ mol/cm^3^)*
5	6.37 ± 0.25	537.87 ± 91.00	9.67	17900.86	500.25	0.76
7.5	5.20 ± 0.05	3583.96 ± 146.06	8.08	11882.31	383.86	1.14
10	4.71 ± 0.03	7262.86 ± 287.04	7.40	9817.134	338.87	1.39

The volumetric swelling ratio (*Q*), average molecular weight between cross-links (*Mc*), average distance between cross-links, that is, mesh size (*ξ*) and network density of the cross-links (*ρ*) were calculated as described in the Supplementary Data S2.

## Discussion

In this study, the impact of Gel-MOD stiffness on chondrogenic and osteogenic differentiation of photo-encapsulated hASC/hTERT microspheroids was investigated, which to our knowledge has not yet been studied. hASC/hTERT have been used, as their differentiation potential has been confirmed to be stable through numerous population doublings.^38^ Cells were encapsulated in 5%, 7.5%, and 10% Gel-MOD (degree of substitution 63%) as this concentration range proved to support long-term proliferation of numerous human and animal cells^8,11,39–46^ and was successfully used for bioprinting applications.^43,47^

The measured mechanical properties of the gels confirmed that increasing concentrations of Gel-MOD drastically enhanced the hydrogel stiffness (i.e. *G*′ from 538 ± 91 up to 7263 ± 287 Pa). While the *G*′ of native cartilage is 400–800 kPa, osteogenesis predominantly occurs in matrices with an elastic modulus of 11–30 kPa,^48–52^ which equals to *G*′ = 3.7–10.7 kPa (“Calculation of the storage modulus from the elastic modulus” section in Supplementary Data S2). As expected, the mechanical properties of the tested hydrogels were much lower than in cartilage, but the stiffness of 7.5% and 10% hydrogels was in range of osteogenesis promotion. Higher Gel-MOD concentrations resulted in a denser network formation, which was calculated using the rubber elasticity theory (Table 2). This is a consequence of longer kinetic oligo-/polymethacrylate chain formation in between the gelatin chains at higher gelatin concentrations, leading to networks with less defects.^35^

Regardless of the hydrogel stiffness or medium used, no shrinkage or degradation of Gel-MOD samples was observed during their 3- to 5-week culture. Gel clots were anchored to the bottom of culture dishes, which prevented floating in the medium and changes in their shape. Previously, the 5% and 10% (w/v) Gel-MOD hydrogels, after incubation with 100 mmol collagenase solution (i.e. 100 collagenase digestion U/mL), proved fully degradable within 77.7 and 210 min, respectively.^9,53^ However, this concentration is incomparably high to the nanomolar concentrations of degrading enzymes found in tissues.^54^ Therefore, we assume that our experimental conditions did not have a major impact on Gel-MOD degradation.

Although the photo-encapsulation process could have damaged the cells, cell viability determined with live/dead staining was preserved in almost all hydrogels. Previous reports confirm that after photo-encapsulation of human MSCs, adipocytes or foreskin fibroblasts within Gel-MOD + Li-TPO or PEG-diacrylate + Li-TPO, cell viability was >90%.^55–57^ In addition, a 23% increase in proliferation of rabbit MSCs was reported after their photo-encapsulation in Gel-MOD + Li-TPO and their 2-week chondrogenic induction.^58^ However, in our study a partial necrosis of microspheroid cores was observed in the 10% hydrogel, cultured for 1 week in the chondrogenic medium. As the microspheroid diameter was ∼200 μm, which was confirmed to support diffusion of oxygen and nutrients, the size of microspheroids was not the cause of apoptosis.^25,59^ Compared to the control or osteogenic medium, chondrogenic medium did not contain serum components (NBCS), as these caused chondrocyte de-differentiation *in vitro*.^60^ The absence of NBCS in the medium could be one reason for lower cell viability and recovery in the 10% Gel-MOD hydrogel. Namely, NBCS is a widely used growth supplement in cell culture and plays a crucial role in attenuating cytotoxic consequences induced by necrotic and apoptotic signals in neuronal cells.^61,62^ As the experiment progressed, the necrotic core gradually disappeared, and cells acquired a rounder morphology, typical of human chondrocytes, which proliferate slowly.^63^

Interestingly, after 3 and 5 weeks of chondrogenic differentiation, “voids” in the cores of the microspheroids were noticed. As this feature was not observed in either control or osteogenically differentiated samples, we conclude that it is a consequence of cell-induced ECM deposition during their 3- to 5-week chondrogenic differentiation. This conclusion is supported by histological results, showing that cells were surrounded with GAGs, major components of the ECM.

After hASC/hTERT microspheroidal encapsulation in 5–10% gels and their culture in control medium, extensive cell sprouting was observed at week 1, which resulted in a complete merging of microspheroids in the 5% hydrogel. This is not surprising, as other cell types are known to spread easily in gelatin-based hydrogels.^8,13,15^ Extensive cell sprouting was observed in the 5% gels cultured in osteogenic medium, this occurred to a lesser extent in stiffer hydrogels, suggesting that the differentiation of cells was favored over their proliferation. A similar observation was reported recently for rat MSCs, which lost their ability to proliferate in Gel-MOD after 14 days of osteogenic induction.^11^

The extent of a 3-week chondro- and osteogenic differentiation of encapsulated hASC/hTERT was evaluated through expressions of genes known to play important roles in chondrogenesis (*SOX9*, *ACAN*, *COL2A1*) and osteogenesis (*RUNX2*, *BGLAP*, *ALPL*, and *COL1A1*) of stem cells.^64,65^ In addition, the expression of a chondrocyte hypertrophic marker *COL10A1* was verified on control and chondrogenically differentiated samples.^66^

Regardless of the hydrogel stiffness, the encapsulated microspheroids cultured in chondrogenic medium expressed extraordinarily high levels of *SOX9*, *ACAN*, and *COL2A1*, which was also confirmed with the calculated differentiation index. These results show that chondrogenically induced hASC/hTERT microspheroids encapsulated in Gel-MOD hydrogels accomplished a high level of chondrogenic differentiation. However, a high expression of *COL10A1* in the samples would suggest that the differentiated cells became hypertrophic. Nonetheless, as the osteogenic marker genes were not simultaneously elevated and the expressions of *SOX9* and *COL2A1* (which are not found in hypertrophic chondrocytes), were extremely high, we assume that this was not the case.^67^ Furthermore, the calcium quantity in these samples was not elevated. Besides, the expression of *COL10A1* was also reported to be present during chondrogenic differentiation of human MSCs and ASCs.^21,68–71^

Histological analysis of hydrogel-tissue cross-sections of chondrogenically differentiated samples showed a strong GAG presence (i.e. positive Alcian blue staining). The color intensity was stronger after 5 weeks of cell differentiation, when structural features of the *in vitro* engineered hydrogel-tissue construct resembled the morphological characteristics of the *in vivo* hyaline cartilaginous tissue.^72^ The extent of chondrogenic differentiation appeared superior in the two softer hydrogels. This could be because of the easier migration of hASC/hTERT within the gel network, which also sustained osteogenic differentiation to a higher degree. A previous publication confirmed that softer agarose gels (modified with Arg-Gly-Asp motifs) exhibited higher DNA and GAG content as well as larger clusters of encapsulated porcine chondrocytes.^73^ Maintenance of better chondrogenic phenotype characteristics was also reported for softer PEG hydrogels.^74,75^

Compared to cells grown in 2D, cells encapsulated in Gel-MOD cultured in control medium showed a moderate expression of *SOX9* and slightly elevated *ACAN* and *COL2A1*, especially in 5% and 7.5% hydrogels. This implies that softer Gel-MOD hydrogels are themselves capable of a slight induction of chondrogenesis. A positive Alcian blue staining was detected in histological sections of control samples of 7.5% and 10% hydrogels, also supporting this assumption.

The analysis of selected osteogenic genes, which have been reported to be expressed in human MSCs after a 3-week or longer osteogenic differentiation, revealed that compared to cells grown in 2D, all Gel-MOD hydrogels containing hASC/hTERT microspheroids cultured in either control or osteogenic medium, expressed moderately higher amounts of *RUNX2*, *BGLAP*, and *ALPL*.^76–78^ However, when these expressions were compared among Gel-MOD control and osteogenic samples, they appeared similar. The same was also observed for *RUNX2* and *BGLAP* in samples cultured in chondrogenic medium. Similarly to our case, slightly higher expressions of *RUNX2* and *ALPL* in chondrogenically differentiated human MSCs were noticed after their encapsulation in alginate gels.^68^ These previous findings and our results indicate that the chondrogenic medium causes a partial upregulation of some osteogenic genes.

By comparing the expression profiles of *RUNX2*, *BGLAP*, *ALPL*, and *COL1A1* it would appear that encapsulated hASC/hTERT cultured in control or osteogenic medium achieved a higher extent of osteogenic differentiation in the 10% Gel-MOD hydrogel. Although the von Kossa staining did not confirm the presence of mineralization in any Gel-MOD control, a strong mineral content was observed throughout the analyzed cross-section of the osteogenically differentiated 5% and 7.5% gels, especially on sites where microspheroids were present. This was expected, as intense localized mineral deposition is a known feature of osteogenically differentiated stem cells within microtissues.^29^ In addition, Alizarin red quantification results confirmed that compared to control Gel-MOD samples, the samples cultured in osteogenic medium contained two or three times more calcium when encapsulated in the 10% hydrogel or the two softer ones, respectively. This would suggest that softer Gel-MOD hydrogels also better support osteogenic differentiation.

Compared to a 10% (w/v) Gel-MOD hydrogel, a favorable osteogenic differentiation of a single cell encapsulation of rat MSCs was recently reported for a 5% hydrogel.^39^ Significant differences in calcium content were observed between the two hydrogel stiffnesses at day 28. Besides, a significantly higher DNA content was detected in 5% hydrogels, which could be the result of a stronger cell attachment as the higher porosity and pore size allowed a higher diffusion of calcium and phosphate ions, leading to a more homogenous calcium deposition throughout the hydrogel.

A stronger chondrogenic versus osteogenic differentiation in the presence of the corresponding differentiation medium could be the result of the chondrogenically favorable condensation state of cells in microspheroids or because of the cell characteristics themselves. Namely, it was shown that distinct stem cell subpopulations isolated from human adipose tissue exhibited different chondrogenic and osteogenic differentiation potential.^79,80^ In our experiments, hASC/hTERT were obtained from one donor whose genetic background could exhibit a better chondrogenic than osteogenic potential.

In this study, the impact of different Gel-MOD stiffnesses (i.e. 5, 7.5, and 10 wt%) on chondrogenic and osteogenic differentiation potential of encapsulated hASC/hTERT microspheroids, cultured for 3–5 weeks in a corresponding differentiation medium, was evaluated. Although all tested hydrogels sustained long-term cell proliferation and survival, both differentiation pathways proved to be well supported by the two softer hydrogels, which better promoted cell migration. The hydrogel-microspheroid strategy proved exceptionally successful in promoting chondrogenesis, which was confirmed at the gene and protein levels. Moreover, Gel-MOD itself showed some potential to direct encapsulated hASC/hTERT microspheroids toward the chondrogenic lineage.

The effects of Gel-MOD on the differentiation of stem cell microspheroids should be explored further, as this hydrogel shows promising potential for future cartilage or bone TE applications.

## Supplementary Material

Supplemental data
