# Supplementary material for: Impact of Hydrogel Stiffness on Differentiation of Human Adipose-Derived Stem Cell Microspheroids
Source: Tissue Eng Part A. 2019 Oct 4;25(19-20):1369–80. doi: 10.1089/ten.tea.2018.0237 (PMC6784494; doi:10.1089/ten.tea.2018.0237)
Supplement: Supplemental data [file Supp_Data.pdf]

Supplementary Data

Supplementary Data S1: Histological and Calcium Quantification Analyses

Histology

Following standard protocols, 2 µm thick sections were deparaffinized and incubated in: (1) Alcian blue solution (pH 2.5; Merck KGaA, Germany) for 30 min and counterstained with nuclear fast red (Merck KGaA) for 3 min, or (2) 1% (w/v) silver nitrate solution (Sigma-Aldrich), placed under UV light for 20 min, washed several times with deionized water (dH_2O), incubated with 5% (w/v) sodium thiosulfate solution (Sigma-Aldrich), and counterstained with nuclear fast red, each for 5 min. Sections were afterward dehydrated through graded alcohol and cleared in xylene before mounting (Supplementary Fig. S1). Images were acquired with the LSM 700 microscope and processed using ZEN 2 core v2.4 software (Zeiss).

Calcium quantification assay

After the Gel-MOD clots were fixed overnight in Roti® Histofix 4% and washed several times with phosphate-buffered saline (1×), they were incubated in 40 mM Alizarin Red S solution for 15 min. After several additional washes with dH_2O, each hydrogel clot was transferred into a 1.5 mL tube, covered with 0.5 mL of 20% methanol/10% acetic solution in dH_2O, and disrupted using a micropestle (Sigma-Aldrich). Samples were centrifuged 4 min at 4000 *g* and 100 µL of each supernatant was transferred in triplicate to a 96-well plate. Absorbance was measured at 450 nm on a Synergy H1 spectrophotometer and calcium content was determined comparatively to the Alizarin Red standards.

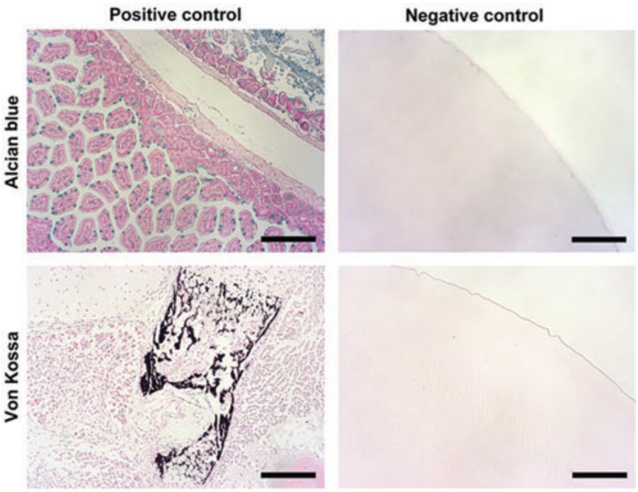

SUPPLEMENTARY FIG. S1. Positive and negative controls of the Alcian blue and Von Kossa staining. *Alcian blue:* positive control—mouse small intestine; negative control—10% Gel-MOD, cultured for 3 weeks in chondrogenic medium. Mucins (glycoproteins) secreted from the goblet cells stained *light blue*. *Von Kossa:* positive control—mouse embryonic tissue; negative control—10% Gel-MOD, cultured for 3 weeks in osteogenic medium. *Black deposits* represent produced calcium minerals. Scale bars = 200 µm.

Supplementary Data 2: Network Density Assessment

Gel-MOD molecular weight determination using Gel permeation chromatography

The molecular weight (MW) of Gel-MOD (with a 63% degree of substitution) was determined on a set-up composed of a Waters 610 fluid unit, a Waters 600 control unit, and a Waters 410 RI detector (Zellik, Belgium). The measurements were performed at 1 mL/min in a 0.1 M phosphate-buffered solution at pH 7.4, using 3 pullulan standards (Shodex, Germany) (i.e. MW = 9890, 21,400, and 276,500 Da) to obtain a calibration curve. Gel-MOD was injected in the phosphate buffer, starting from the initial solution of 1 mg/mL (Supplementary Fig. S2A).

Network density calculations using the rubber elasticity theory

An estimation of the actual network density (ρ), the mesh size (ξ), and the average MW between cross-links (Mc) can be calculated using the rubber elasticity theory from the mass swelling ratio, the rheological results and the original MW.[S1,S2] However, to calculate these variables, the volumetric swelling ratio (Q) has to be first calculated from the mass swelling ratio (q). This can be done using Equation (S1), where $v_{2,s}$ is the polymer volume fraction in the swollen state, V_p and V_g are the polymer and hydrogel volume at equilibrium swelling, respectively, and ρ_{H_2O} and $\rho_{gelatin}$ are the densities of water and gelatin, respectively (i.e. 1 and 1.36 g/cm^3).[S1–S4]

$$v_{2,s} = \frac{V_p}{V_g} = \frac{1}{Q} = \frac{\left(\frac{1}{\rho_{\text{gelatin}}}\right)}{\left(\frac{q}{\rho_{\text{H}_2\text{O}}}\right) + \left(\frac{1}{\rho_{\text{gelatin}}}\right)}. \tag{S1}$$

To calculate the average distance between cross-links from the volumetric swelling ratio (Q), Equation (S2) can be applied under the condition that all network chains within the characterized hydrogels follow the Gaussian statistics model as evidenced by a linear correlation between log G and log Q for all samples (Supplementary Fig. S2B).[S5,S6]

$$G = \left(\frac{cRT}{\overline{Mc}}\right) \times \left(1 - \frac{2\overline{Mc}}{M_n}\right) \times \left(\frac{1}{Q^{1/3}}\right). \tag{S2}$$

In Equation (S2), G represents the shear modulus (atm), c is the concentration of gelatin in the solution, R is the universal gas constant ($L \times \text{atm} \times K^{-1} \times \text{mol}^{-1}$), T is temperature (K), $\overline{Mc}$ is the average MW between cross-links (Da), and M_n is the numerical MW of Gel-MOD before cross-linking (see Supplementary Fig. S2). The shear modulus can be

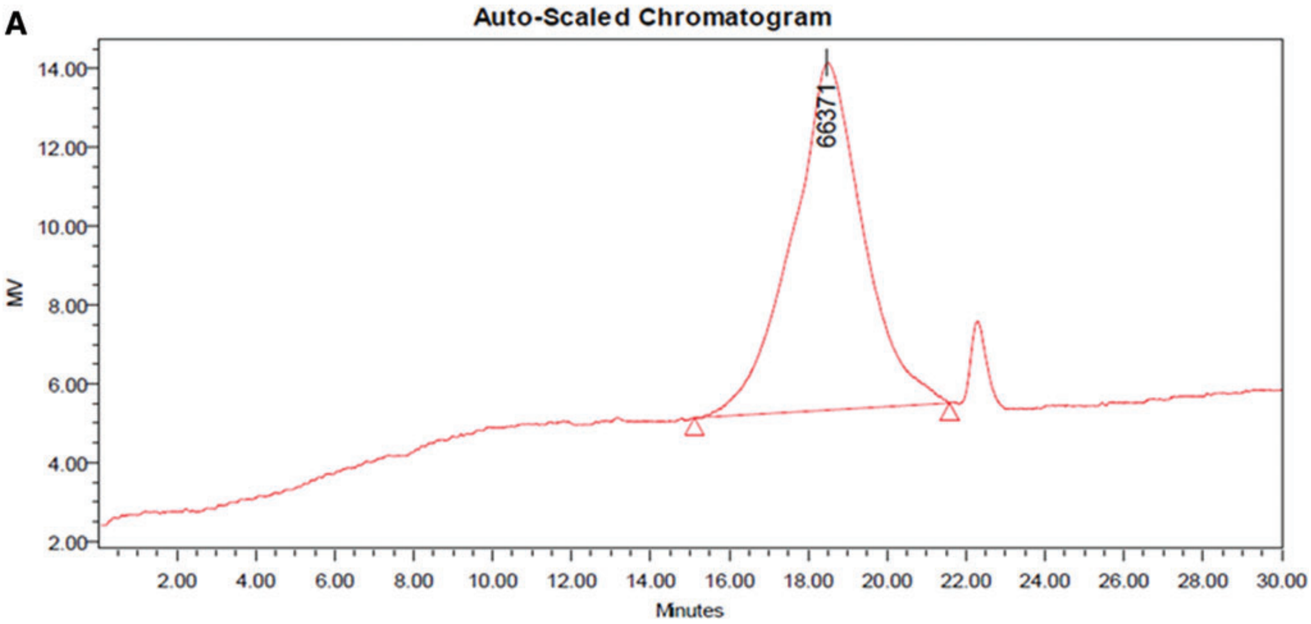

GPC Results

	Dist Name	Mn	Mw	MP	Mz	Mz+1	Mv	Polydispersity	MW Marker 1	MW Marker 2
1		42575	80152	66371	129571	170909		1.882617		

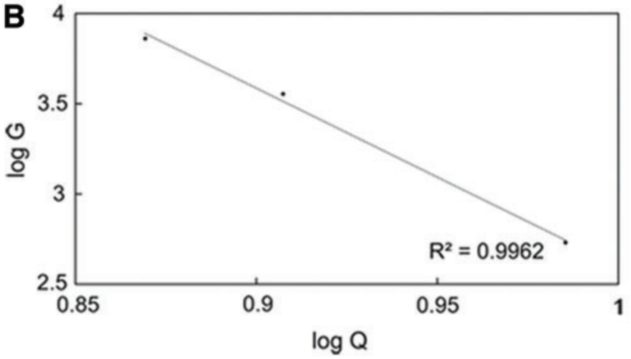

SUPPLEMENTARY FIG. S2. **(A)** GPC of Gel-MOD, with MW calculations (M_n, M_w, and polydispersity) according to pullulan standards. **(B)** Plot of log G versus log Q for all analyzed hydrogel films evidencing a Gaussian distribution. GPC, gel permeation chromatogram; MW, molecular weight.

derived from the mean peak value of the storage modulus because the contribution as the loss modulus G'' to the shear modulus can be considered negligible in comparison with the storage modulus for all analyzed samples.[S2,S7,S8]

Using the obtained average MW between cross-links, an estimation of the average mesh size (ξ) in equilibrium swollen state can be calculated using Equation (S3)[S9]:

$$\xi = \left(\frac{2C_n\overline{Mc}}{M_r}\right)^{(1/2)} \times l \times Q^{(1/3)}. \qquad \text{(S3)}$$

Here C_n represents the Flory characteristic ratio, corresponding to 8.26 for gelatin,[S9] M_r is the average MW of one repeating unit, or one amino acid (on average ~94.7 g/mol[S9,S10]) and l corresponds to the length of a bond along the polymer backbone. In this case one repeating unit corresponds to 1 C–C (carbonyl bond) (i.e. 1.53 Å) and the mean between a C–N (i.e. 1.47 Å) and a C(carbonyl)–N bond (i.e. 1.32 Å) or 2.925 Å.[S9,S11] Furthermore, the equation is based on the Flory–Rehner theory for simple vinyl polymers, which is not the case for peptides. Therefore a factor 2 has to be replaced by a factor 3 since the repetitive unit contains two bonds in contrast to one bond in vinyl polymers.[S9] Therefore, the equation can be rewritten as in Equation (S4):

$$\xi = \left(\frac{3C_n\overline{Mc}}{M_r}\right)^{(1/2)} \times l \times Q^{(1/3)}. \qquad \text{(S4)}$$

Finally, the cross-link density (ρ_x) represents the number of cross-links as a function of the volume, which can be calculated from $\overline{Mc}$ and $\overline{\nu}$, where $\overline{\nu}$ corresponds to the specific volume of gelatin (i.e. 0.735 cm^3/g), as given in Equation (S5).[S2]

$$\rho_x = \frac{1}{\bar{v}\overline{Mc}}. \quad \text{(S5)}$$

Calculation of the storage modulus from the elastic modulus

Considering materials as ideal rubbers, which is the case of hydrated hydrogels, it is possible to estimate the storage modulus from an elastic modulus value using Equation (S6)[S12]:

$$G' = \frac{E'}{2\,(1 + \mu)}. \quad \text{(S6)}$$

In this equation G' represents the shear storage modulus (Pa), E' represents the elastic modulus (Pa), and μ the Poisson number, which in the case of ideal rubbers equals to 0.5.

References

S1. Van Hoorick, J., Gruber, P., Markovic, M., *et al.* Cross-linkable gelatins with superior mechanical properties through carboxylic acid modification: increasing the two-photon polymerization potential. Biomacromolecules **18**, 3260, 2017.

S2. Billiet, T., Gasse, V.B., Gevaert, E., Cornelissen, M., Martins, J.C., and Dubruel, P. Quantitative contrasts in the photopolymerization of acrylamide and methacrylamide-functionalized gelatin hydrogel building blocks. Macromol Biosci **13**, 1531, 2013.

S3. Veis, A. The Macromolecular Chemistry of Gelatin. New York: Academic Press, 1964.

S4. Ganji, F., Vasheghani-Farahani, S., and Vasheghani-Farahani, E. Theoretical description of hydrogel swelling: a review. Iran Polym J **19**, 375, 2010.

S5. Van Vlierberghe, S., Dubruel, P., Lippens, E., Cornelissen, M., and Schacht, E. Correlation between cryogenic parameters and physico-chemical properties of porous gelatin cryogels. J Biomater Sci Polym Ed 2**0**, 1417, 2009.

S6. Lee, K.Y., Bouhadir, K.H., and Mooney, D.J. Controlled degradation of hydrogels using multi-functional cross-linking molecules. Biomaterials **25**, 2461, 2004.

S7. Mironi-Harpaz, I., Wang, D.Y., Venkatraman, S., and Seliktar, D. Photopolymerization of cell-encapsulating hydrogels: crosslinking efficiency versus cytotoxicity. Acta Biomater **8**, 1838, 2012.

S8. Kuijpers, A.J., Engbers, G.H.M., Feijen, J., *et al.* Characterization of the network structure of carbodiimide cross-linked gelatin gels. Macromolecules **32**, 3325, 1999.

S9. Ma, S., Natoli, M., Liu, X., *et al.* Monodisperse collagen–gelatin beads as potential platforms for 3D cell culturing. J Mater Chem B **1**, 5128, 2013.

S10. Mark, J.E., ed. Polymer Data Handbook. New York, NY: Oxford University Press, 1999.

S11. Corey, R.B., and Pauling, L. Fundamental dimensions of polypeptide chains. Proc R Soc B **141**, 10, 1953.

S12. Meyvis, T.K.L., Stubbe, B.G., Van Steenbergen, M.J., Hennink, W.E., De Smedt, S.C., and Demeester, J. A comparison between the use of dynamic mechanical analysis and oscillatory shear rheometry for the characterisation of hydrogels. Int J Pharm **244**, 163, 2002.
